# A contemporary understanding of iron metabolism in active premenopausal females

**DOI:** 10.3389/fspor.2022.903937

**Published:** 2022-07-28

**Authors:** Claire E. Badenhorst, Adrienne K. Forsyth, Andrew D. Govus

**Affiliations:** ^1^School of Sport, Exercise, and Nutrition, College of Health, Massey University, Auckland, New Zealand; ^2^School of Behavioural and Health Science, Australian Catholic University, Fitzroy, VIC, Australia; ^3^Discipline of Sport and Exercise, Department of Sport, Exercise, and Nutrition Science, La Trobe University, Melbourne, VIC, Australia

**Keywords:** menstrual cycle, menstruation, hepcidin, iron regulation, iron status

## Abstract

Iron metabolism research in the past decade has identified menstrual blood loss as a key contributor to the prevalence of iron deficiency in premenopausal females. The reproductive hormones estrogen and progesterone influence iron regulation and contribute to variations in iron parameters throughout the menstrual cycle. Despite the high prevalence of iron deficiency in premenopausal females, scant research has investigated female-specific causes and treatments for iron deficiency. In this review, we provide a comprehensive discussion of factors that influence iron status in active premenopausal females, with a focus on the menstrual cycle. We also outline several practical guidelines for monitoring, diagnosing, and treating iron deficiency in premenopausal females. Finally, we highlight several areas for further research to enhance the understanding of iron metabolism in this at-risk population.

## Introduction

Research in exercise-related iron deficiency has transitioned from treatment-focused research that primarily addresses dietary iron intake and supplement effectiveness, to a more prevention-focused approach that has addressed causes of iron deficiency and the influence of the iron regulatory hormone, hepcidin, on iron homeostasis (Nemeth et al., 2004; Peeling et al., 2009; Ganz, 2011; Pasricha et al., 2011; Sim et al., 2019). Iron deficiency is particularly prevalent in active females (~15–50%) (Fallon, 2004; Parks et al., 2017) compared with their less active counterparts (~10–14%) (Marx, 1997; WHO, 2019). The increased prevalence of iron deficiency in active females is associated with adverse health outcomes including fatigue, poor mood, decreased cognitive function, increased risk of illness and injury, impaired thermoregulation, and reduced exercise tolerance (Pasricha et al., 2010; Sim et al., 2019). Identified contributors to exaggerated iron losses and inadequate iron intakes include low total dietary iron intake and/or bioavailability (Craig, 1994; Castell et al., 2019), dietary intake patterns, iron lost through menstrual blood loss (Napolitano et al., 2014; Bruinvels et al., 2016), and exercise-related mechanisms (i.e., sweating, hemolysis, gastrointestinal bleeding). In addition, increased hepcidin activity ~3–6 h after exercise may induce a transient reduction in iron recycling and absorption thereby reducing the uptake of iron from iron rich meals consumed post-exercise. This is likely to increase a premenopausal female athlete's risk of developing an iron deficiency if they are unable to replenish their daily iron losses (Peeling et al., 2009; Sim et al., 2019).

Given hepcidin's crucial role in controlling iron uptake kinetics after exercise, research has sought to clarify how lifestyle factors (e.g., dietary intake/patterns) (Badenhorst et al., 2015a,b, 2016; McKay et al., 2019, 2020) and external environments (e.g., altitude and heat) (Badenhorst et al., 2014; Govus et al., 2017; Hayashi et al., 2020; McKay et al., 2021; Sumi et al., 2021) influence hepcidin kinetics in active individuals. Research has also investigated the changes in hepcidin kinetics in response to exercise training, the timing of iron-rich foods and oral iron supplements which has provided translatable research outcomes detailing when iron could be ingested by active individuals to enhance its uptake (Moretti et al., 2015; Stoffel et al., 2017; McCormick et al., 2019, 2020).

Most iron metabolism research in active populations has been conducted in male-only or mixed cohorts. Consequently, few guidelines (Pedlar et al., 2018; Sim et al., 2019) provide nuanced recommendations about how to manage iron status in active premenopausal females. Furthermore, only two investigations to date have measured changes in the activity of the master iron-regulatory hormone, hepcidin, throughout the menstrual cycle (Angeli et al., 2016; Lainé et al., 2016), and only one research program has investigated changes in hepcidin activity acutely after exercise in premenopausal females (Peinado et al., 2021). This clear lack of research into understanding the regulation of iron parameters and hepcidin in active premenopausal females throughout their menstrual cycle has limited the creation of clear practical recommendations to improve the diagnosis, management, and treatment of iron deficiency in this at risk cohort. This review therefore discusses iron status and regulation from a female-specific perspective, examining the changes in iron parameters and hepcidin kinetics that are expected throughout the menstrual cycle in eumenorrheic premenopausal females (i.e., defined as natural menstruating females, with menstrual cycles that are 21–35 days, exhibiting a luteinizing hormone surge and a correct hormone profile with no influence from exogenous hormones for >3 months; Elliott-Sale et al., 2021) or premenopausal females presenting with menstrual dysfunction (i.e., anovulation or luteal phase defects). Finally, we provide practical recommendations for sports scientists, health and medical practitioners, to guide the diagnosis and treatment/s of iron deficiency in premenopausal females.

## Iron requirements for active females

Iron is an essential dietary nutrient that forms the functional component of several heme and non-heme proteins involved in oxygen transport (hemoglobin), oxygen storage (myoglobin), and energy production *via* oxidative and glycolytic enzymes (Beard et al., 1996). For iron to support physiological functions, the primary function being erythropoiesis, iron absorption from dietary iron intake must balance daily iron losses (Beard and Han, 2009). In sedentary individuals, ~2–3 mg of iron is absorbed daily from dietary iron intake to replenish iron losses and maintain iron homeostasis (Beard and Han, 2009). In eumenorrheic females, menstrual bleeding largely contributes to iron losses (Arens, 1945; Hallberg and Nilsson, 1964), with ~1 mg of iron lost per day during menstrual bleeding (Hallberg et al., 1966). In active females, an additional 3–4 mg/day of iron may be required to replenish exercise-related iron losses (e.g., hemolysis, hematuria, gastrointestinal bleeding, sweating, and dermal losses) (Nielsen and Nachtigall, 1998). The iron demands of exercise, in addition to daily iron losses and menstruation, may result in a negative iron balance, which if not compensated for by either dietary means and/or iron supplementation, increases the risk of iron deficiency with or without anemia in active premenopausal females.

### A brief overview of systemic iron regulation: The role of hepcidin

Systemic iron homeostasis is coordinated by the body's iron regulatory hormone, hepcidin, a 25-amino acid peptide hormone that is produced by the liver (Hare, 2017). Hepcidin inhibits excessive systemic iron levels by internalizing and proteolytically degrading the body's only known iron transporter, ferroportin, which is expressed on the basolateral surfaces of reticuloendothelial macrophages in the spleen, liver, and enterocytes (Hare, 2017). Hepcidin also acts on divalent metal transporter-1 (DMT-1) channels on the apical surface of duodenal enterocytes to limit intestinal iron absorption (Brasselagnel et al., 2011). When hepcidin levels are high, less iron can combine to apotransferrrin and be transported as transferrin (an iron transport glycoprotein) around the body to target cells such as erythroid precursor cells in the bone marrow, which support erythropoiesis. Several mechanisms influence hepcidin activity *in vivo*, including factors directly related to iron status and iron utilization within the body, such as plasma iron concentration, iron stores (ferritin), and the rate of erythropoiesis (Nemeth and Ganz, 2009). Additionally, hepcidin expression increases in response to infection and inflammation as part of the body's acute phase response, with the cytokine Interleukin-6 (IL-6) the main stimuli responsible for limiting iron availability for bacterial growth and viral replication (Ganz and Nemeth, 2015). The increase in hepcidin activity in response to inflammation has been identified as a potential contributor to altered iron utilization in active individuals (Peeling et al., 2009). Research in iron replete individuals (serum ferritin >35 μg/L; (Sim et al., 2019) has consistently demonstrated that elevations in IL-6 concentration immediately after exercise, in an intensity- and duration-dependent manner, promotes an increase in hepcidin activity ~3–6 h following exercise cessation (Peeling et al., 2014). Altered iron utilization (recycling and absorption) kinetics post-exercise potentially align with post-exercise dietary intake, and in combination with the previously mentioned iron losses may jeopardize iron status in active females, increasing their risk of iron depletion and iron deficiency diagnosis.

Several other regulators of hepcidin activity have been identified including growth factors (e.g., myonectin) (Goodnough et al., 2012; Halon-Golabek et al., 2019), the mechanistic target of rapamycin (mTOR) (Guan and Wang, 2014), testosterone (Bachman et al., 2010), estrogen (E2) (Hamad et al., 2020), progesterone (P4) (Li et al., 2015), growth hormone (Vihervuori et al., 1994), leptin (Yamamoto et al., 2018), and insulin (Fillebeen et al., 2020). Since female sex hormones (progesterone and estrogen) regulate hepcidin activity, we next discuss the influence of female sex hormones on hepcidin and systemic iron metabolism.

## The influence of sex hormones on iron regulation

The menstrual cycle is defined by biphasic fluctuations in E2 and P4, with each cycle lasting approximately 28 days, but may range from 21 to 35 days (Elliott-Sale et al., 2021). Estrogen, and in particular 17β-estradiol (E2), is the most abundant endogenous form of E2 in human females. Estrogen is considered the primary female sex steroid, and elevated concentrations of E2 have been positively correlated with iron demand and the release of iron into the systemic circulation, and negatively correlated with hepcidin concentration (Hamad et al., 2020), however, the exact mechanism by which E2 influences iron regulation is still to be determined. *In vitro* studies in breast (Bajbouj et al., 2018), ovarian SKOV3 (Yang et al., 2012), liver cells (HUH7 and Hep-G2) (Hou et al., 2012) and rodent models suggest E2 may support the upregulation of genes involved in iron metabolism (e.g., ferroportin, lactotransferrin, ferroxidase ceruloplasmin, lipocalin 2) (Stuckey et al., 2006; Bajbouj et al., 2018; Hamad et al., 2020), likely by downregulating hepcidin activity. In ovarian cells (E2-S and SKOV-3), E2-induced upregulation of HIF-1α has also been shown to downregulate hepcidin gene (*HAMP)* expression, subsequently reducing hepcidin concentration (Hou et al., 2012). Additionally, in human liver cells treated with E2, reduced hepcidin levels were observed to occur due to the binding of E2 on E2 responsive elements (ERE) on the *HAMP* gene, suppressing the *HAMP* gene expression (Hou et al., 2012). *In vivo* studies have demonstrated a marked suppression in serum hepcidin levels from 4.85 to 1.43 ng/mL (a decline of ~40%) (Lehtihet et al., 2016) in females treated with large doses of E2 (0.15 to 3.99 ng/mL an E2 increase of ~25%) during *in vitro*-fertilization (IVF).

While most available research suggests an inverse relationship between E2 and hepcidin, two studies have reported an increase in *HAMP* gene expression and hepcidin synthesis in response to E2 treatment (Ikeda et al., 2012; Bajbouj et al., 2020). In ovariectomized mice, E2 appeared to increase serum and liver iron through increased *HAMP* gene expression which occurred *via* a GPR30 (G-protein coupled receptor 30), the 7-transmembrane E2 receptor (Ikeda et al., 2012). Further, treatment with E2 and G1 (GPR30 agonist) reduced BMP6 and hepcidin expression, suggesting E2 may be involved in hepcidin expression *via* a GPR30-BMP6-dependent mechanism (Ikeda et al., 2012). The response of E2 on *HAMP* gene expression may thus depend on the differential expressions of E2 receptors, such as membrane-anchored E2 receptors GPR30, and their co-regulators in various cell types (Ikeda et al., 2012).

The relationship between E2 and iron homeostasis may be cell-type specific. In monocytes, E2 may differentially alter iron metabolism in an IL-6-dependent manner. *In vitro* exogenous treatment with E2 in human cell lines, including U937 cells, initiated a cascade of effects commencing with an increase in IL-6 synthesis, a reduction in TNF-α, HIF-1α, and ERα gene expression followed by an increase in hepcidin levels (Bajbouj et al., 2020). However, in uterine cells, E2 in uterine cells, E2 may play an important role in reducing hepcidin expression and supporting iron turnover during E2-induced cell growth and development (i.e., in the proliferation phase that occurs during the mid-late follicular phase of the menstrual cycle, days 5–14). Conversely, in immune cells, increases in E2 mid-cycle may enhance the pro-inflammatory response in macrophages and dendrites, increasing hepcidin and iron sequestration as part of the anti-inflammatory response (Bajbouj et al., 2020; Hamad et al., 2020).

Two research publications to date have monitored changes in basal hepcidin concentration throughout the menstrual cycle (Angeli et al., 2016; Lainé et al., 2016). During the early follicular phase, defined by the presence of menstrual bleeding and low sex hormone concentrations (low E2 and P4), hepcidin levels appear at their lowest (Lainé et al., 2016). During this phase, iron is actively lost through menstrual bleeding, thus low hepcidin levels during the early follicular phase may reflect the regulation of hepcidin *via* systemic iron levels, compensating for menstrual iron loss by facilitating a physiological state that supports efficient recycling of iron and iron uptake from the diet. This proposal is supported by total iron-binding capacity (TIBC) being at its highest (Kim et al., 1993) during the early follicular phase. There is a graded increase in E2 after menstruation, produced by the developing follicle in the ovaries, that peaks in the late follicular phase (days 10–14 at ~200–250% above early follicular baseline E2 levels) before ovulation in an eumenorrheic female (Constantini and Hackney, 2013; Prior, 2018). Hepcidin levels rebound mid-cycle, which may align with the drop in E2 following the pre-ovulation surge. Additionally, during mid-cycle ovulation, a peak in testosterone levels (increase by ~40%) has been observed in eumenorrheic females (Cook et al., 2021). Testosterone has consistently been shown to potently suppress hepcidin in both males and females (Bachman et al., 2010; Latour et al., 2014). Rodent models demonstrate that hepcidin down-regulation by testosterone occurs due to testosterone-dependent upregulation of epidermal growth factor (EGF) receptors in the liver (Latour et al., 2014). In both younger (19–35 years) and older (59–75 years) males, testosterone suppresses hepcidin in a dose-dependent manner, with these changes being strongly associated with increases in hemoglobin and hematocrit (Bachman et al., 2010). Testosterone levels in females range from 0.4–2.0 nmol/L, ~4–5 times lower than the concentration in men (Kanakis et al., 2019). Females with excess androgens, as seen in Polycystic Ovarian Syndrome (PCOS), have testosterone levels above the physiological range in healthy females (~0.34–5.5 nmol/L), although their testosterone concentrations are still less than age-matched males (Kanakis et al., 2019). A mild iron overload is a common comorbidity in females with PCOS, possibly due to a testosterone-dependent suppression of hepcidin and a large influx of iron into the systemic circulation (Escobar-Morreale, 2012). However, in eumenorrheic females, the suppressive effect of testosterone and E2 on *HAMP* gene expression in some cell types may result in a progressive increase in systemic iron levels during the mid-late follicular phase. After ovulation, the drop in both E2 (E2 drops to ~100% above baseline early follicular E2 levels) and testosterone (which are hepcidin suppressors), coinciding with the increase in systemic iron levels before ovulation may contribute to the rebound in hepcidin during the latter half of the cycle.

Progesterone is the other key steroid sex hormone that is produced by the corpus luteum in a pulsatile manner. Peaks in P4 occur in the luteal phase following successful ovulation, with concentrations of P4 being roughly over 1,000% compared to baseline P4 in the early follicular phase (Constantini and Hackney, 2013; Prior, 2018). Despite P4 being the primary hormone of the luteal phase in eumenorrheic premenopausal females, the effect of P4 on iron metabolism has not been examined in detail. *In vitro* studies have identified P4 as a hepcidin-inducing steroid (HIS) that increases hepcidin biosynthesis in human hepatoma (Hep-G2) cells, independent of inflammation (Li et al., 2015). *In vivo* results in females undergoing IVF who received daily intramuscular injections of 50 mg of P4 had 3-fold and 2-fold higher hepcidin levels on days 6 and 15 of P4 treatments respectively (Li et al., 2015). The induction of hepcidin *via* the P4-mediated pathway appears to be delayed compared to the fast-acting BMP-signaling pathway. Increased hepcidin expression occurred ~4 h following the peak in P4 concentration and peaked ~12 h following the commencement of P4 administration. The delayed rise in hepcidin levels following exogenous P4 administration may suggest that HIS require a secondary intracellular messenger or metabolite to reach a critical threshold before triggering downstream effects that result in increased hepcidin expression. Within the luteal phase, lower TIBC and higher hepcidin levels may be indicative of stable iron utilization (Kim et al., 1993; Angeli et al., 2016; Lainé et al., 2016). These changes may be due to the direct influence of P4 on hepcidin activity, or following the rebound in systemic iron levels around ovulation, hepcidin levels may increase to regulate iron homeostasis and prevent iron excess (Nemeth and Ganz, 2009; Li et al., 2015). With a single research study examining the effects of P4 on hepcidin activity, further research is needed to clarify the *in vivo* effects of P4 on hepcidin levels in eumenorrheic females.

Follicle Stimulating Hormone (FSH) and Luteinizing Hormone (LH) are two hormones that are part of the reproductive axis in both males and females. In females, FSH peaks in the late luteal phase, stimulating the recruitment and development of ovarian follicles in the ovary (Reed and Carr, 2018). Within granulosa cells in the ovary, FSH stimulates aromatase enzyme that aids the conversion of androgens to E2. FSH levels drop at the start of menses as a result of the negative feedback from E2 and inhibin B produced by the developing follicle in the ovary (Groome et al., 1996). The decline in FSH in the early follicular phase is associated with an increase in the androgenic environment within the ovary (Reed and Carr, 2018). During the follicular phase, the increase in size in the dominant follicle and granulosa cells supports the graded increase in E2 from the mid to late follicular phase. In the presence of increasing E2 in the follicular phase, FSH stimulates the formation of LH receptors on the granulosa cells, which results in the secretion of small quantities of P4 and 17-hydroxyprogesterone (17-OHP), both of which have a feed-forward effect on the pituitary gland to start secreting LH. Within the ovary, LH initiates E2 production *via* the conversion of androstenedione to E2 in the thecal and granulosa cells. To a lesser degree, LH also stimulates the production of testosterone in theca cells (Reed and Carr, 2018). LH levels are low during the early follicular phase but will begin to rise in the mid-late follicular phase due to the positive feedback of E2 on the pituitary gland. For LH to be released from the pituitary gland and initiate ovulation, E2 needs to surge above a threshold of 200 pg/mL for 50 h. LH is secreted by the anterior pituitary in a pulsatile manner, with pulses roughly occurring every 60–90 min at a stable amplitude in the early follicular phase, which then increases in frequency and amplitude toward ovulation (Reame et al., 1984). To date, no research has investigated whether FSH or LH exert any influence over *HAMP* gene expression or hepcidin activity. However, the activity of these two gonadotrophic hormones is intrinsically linked to the production of E2 and P4 throughout the menstrual cycle in naturally menstruating females. Therefore, their effect on hepcidin activity and iron regulation is likely to be indirect and associated with the production of E2 and P4.

**Figure 1:**
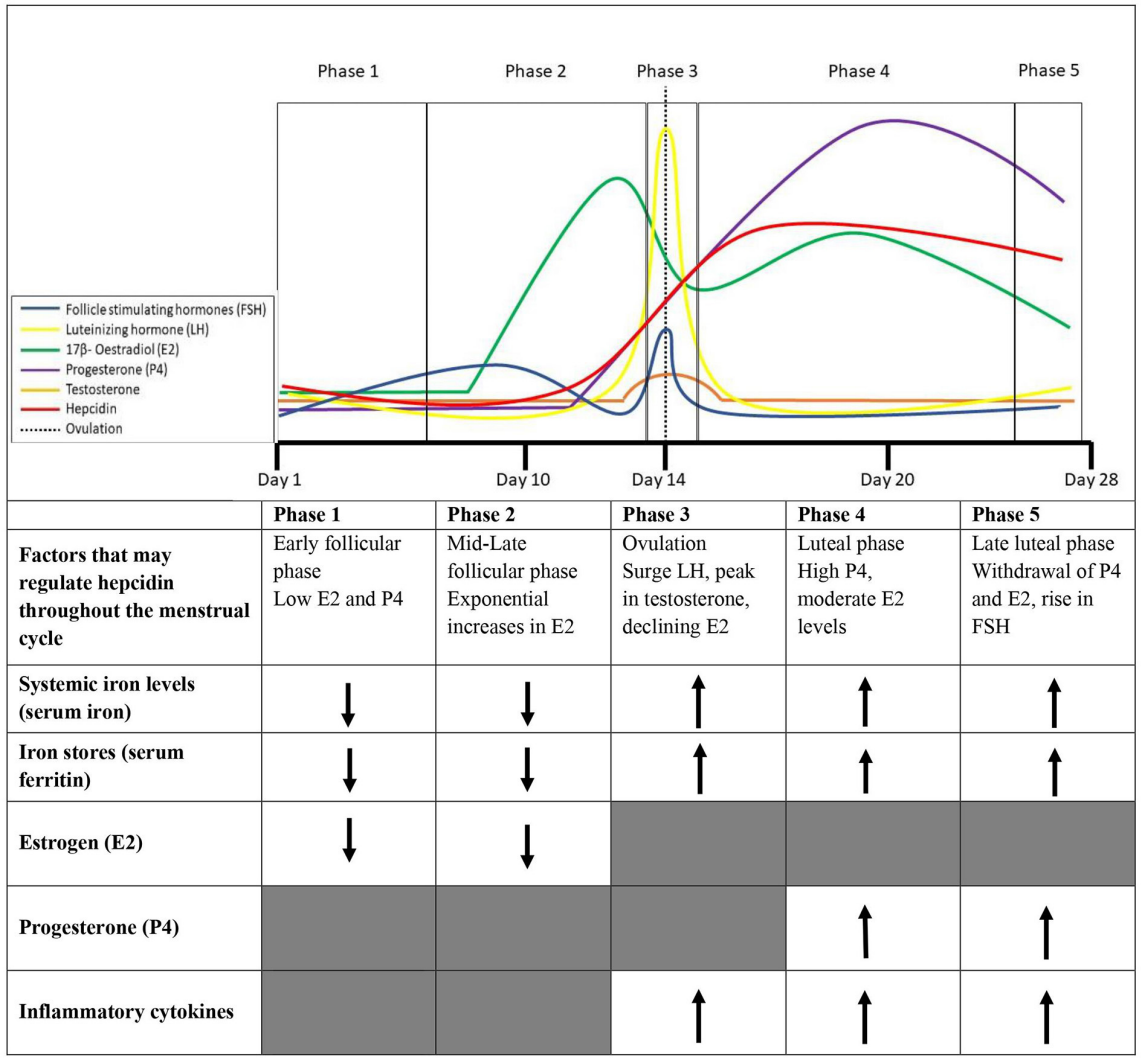
Phases of the menstrual cycle and factors that collectively may contribute to the variability in hepcidin expression throughout the menstrual cycle in healthy eumenorrheic iron sufficient (serum fcrritin > 35 μg/L) females.

Hepcidin activity throughout the menstrual cycle in eumenorrheic premenopausal females and its alignment with sex steroid hormones (E2, P4, and testosterone) and their subsequent influence on systemic iron levels requires further clarification. An overview of sex hormone changes throughout the menstrual cycle and the influence on hepcidin and iron parameters discussed in this sector are presented in Figure 1.

## Inflammation throughout the menstrual cycle

The ovaries and endometrium display repeated inflammation throughout the menstrual cycle, with increases in inflammatory cytokines typically occurring at ovulation and upon the commencement of menstruation (Maybin and Critchley, 2015). Within premenopausal females, the withdrawal of P4 initiates menstrual bleeding and acute inflammatory changes including an influx of leucocytes, an increase in inflammatory cytokines, prostaglandins, and destructive enzymes of the extracellular matrix (Hapangama and Bulmer, 2016). In premenopausal females, ~2-fold increases in IL-6 levels have been observed during menses, with a lesser increase occurring around ovulation (Whitcomb et al., 2014).

Estrogen has a minor influence on lymphocyte proliferation, with some research showing increases in E2 within the menstrual cycle are accompanied by increases in IL-6 (Angstwurm et al., 1997), while others suggest an inverse relationship between E2 and IL-6 (Whitcomb et al., 2014). This inverse relationship may be explained by the timing of data collection that may have aligned with a drop in E2 at ovulation and at the start of menstruation after stimulating IL-6 production. Conversely, P4 has been shown to downregulate cytokine synthesis (Angstwurm et al., 1997), with research consistently demonstrating an inverse relationship between P4 and IL-6 (Whitcomb et al., 2014). The proinflammatory cytokine IL-1β, also observed to have a key role in the progression of inflammation and hepcidin activity (Cannon and Dinarello, 1985; Kanamori et al., 2019), increases post-ovulation during the luteal phase in premenopausal females. The post-ovulation increase of IL-1 from macrophages is potentially due to increased inflammation following follicle rupture and may be induced by E2 and P4 increases in the luteal phase (Cannon and Dinarello, 1985). The changes in inflammatory cytokines do not appear to align with changes in hepcidin in the follicular phase of the menstrual cycle. Hepcidin regulation in the early-late follicular phase may be the result of changes in systemic iron levels (declines with menstrual bleeding), E2 and testosterone activity (peaks before ovulation). During ovulation, elevated systemic iron levels (cessation of menstrual bleeding), declines in E2, and increases in proinflammatory cytokines may contribute to the rebound in hepcidin activity mid-cycle (Figure 1). Whilst during the luteal phase, elevated systemic iron levels, and P4 could contribute to the observed elevated hepcidin levels (Hapangama and Bulmer, 2016).

During the late luteal phase, ~81.1% of females are likely to present with premenstrual syndrome (PMS), which may be induced by, and increase, in response to: inflammatory cytokines, the reduction in reproductive hormones (P4 and E2), or the increase in prostaglandins (Bruinvels et al., 2021). Typical PMS symptoms include fatigue, headaches, mood changes, sleep disruption, and poor concentration/memory (Bruinvels et al., 2021), all of which are commonly associated symptoms with iron deficiency. The association between the severity of PMS and iron stores has not been investigated, and future research may seek to clarify if PMS symptoms are exacerbated when females present with depleted iron stores. Ferritin and hepcidin are both considered acute phase reactants, yet research to date has not clarified if there is an increase in either of these iron parameters in the ~3–5 days before menstruation. With large between-female variability and low within-female variability in inflammation throughout the menstrual cycle (Whitcomb et al., 2014), practitioners should be mindful of the timing of iron parameter collection in females and be sure to collect markers of inflammation to ensure the correct determination of iron status in active females.

## Changes in iron parameters throughout the menstrual cycle in eumenorrheic females

To the best of the authors' knowledge, eight studies have measured the changes in iron parameters and regulation throughout the menstrual cycle (Table 1), albeit with high intra- and inter-individual variability. Serum iron and transferrin saturation have both been reported to be lower in the early follicular phase compared to the mid-late follicular phase and mid-luteal phase in a group of premenopausal females with stage 1 iron deficiency (serum ferritin of <35 μg/L) (Alfaro-Magallanes et al., 2022). Collectively, this prior research has reported decreased serum iron, transferrin, and occasionally hemoglobin, and transferrin saturation (Kim et al., 1993) during menses, followed by a gradual increase in these iron parameters during the mid-to-late-follicular phase, and a plateau during the luteal phase, mirroring the changes in hepcidin observed throughout the cycle.

**Table 1:** Studies that have investigated iron status and hepcidin at different phases of the menstrual cycle in premenstrual females.

**Authors**	**Study ranking for methodological control in female cohorts (Smith et al., 2022)**	**Population**	**Age**	**Blood markers measured**	**Iron deficiency prevalence**	**Measure of the menstrual cycle**	**Comments**
Zilva and Patston (1966)	Bronze	*n* = 11	22–38 years	SI	Not noted	Mean values for SI calculated for each day of the cycle, excluding readings for 6 days around the onset of the menstrual cycle. Average deviation from the mean of SI was calculated for each day of the cycle	Fall in SI 2–3 days into menses, lowest point reached 3rd day of menses. SI increased on the 4th day of the cycle and reached mean SI values around ovulation (~ day 14). Increased slightly and stabilized in the luteal phase.
Kim et al. (1993)	Bronze	*n* = 1,712	18–44 years	Hb, MCV, SI, TIBC, TS, EP, SFer Analyzed in 1/3rd of the females	Defined by SFer and MCV models. SFer model: highest incidence iron deficiency in menstrual phase vs luteal phase (23 vs 8%) MVC model: iron deficiency prevalence decreases with each successive phase of the menstrual cycle. Highest in menstrual (11%) vs luteal (4%) and late luteal (4%)	Collected by interview: • When did you last period or menstrual cycle end? Phases of cycle were operationally defined as: • Menstrual phase: currently menstruating at time of survey • Follicular phase: days 1–9 after menstruation ended • Luteal phases: days 10–16 after menstruation ended • Late Luteal phase: days 17–30 after menstruation ended	Hb, SI and TS values significantly associated with menstrual phase. Lowest in the menstrual phase and highest in luteal phase TIBC: highest in menstrual phase and lowest luteal phase EP: highest menstrual and follicular phases and lowest in luteal and late luteal phase SFer: highest in luteal and late luteal phase MCV: slightly lower in follicular phase vs menstrual phase Serum albumin (hemodilution): higher luteal phase.
Lainé et al. (2016)	Ungraded	*n* = 90	18–45 years	Hb, TS, Fer, SI, SH	29% presented with low iron stores in menses	Serum samples at 6 time points within a cycle: Day 0: start menses 3 visits during menses and in following day Last 2 visits toward the end of the cycle	SH, SI and TS dropped during menses, increasing mid cycle and stabilizing toward the end of the cycle. Higham Score (intensity of blood loss in menses) positively correlated with magnitude of SH and iron variations
Belza et al. (2005)	Bronze	*n* = 13	23–30 years	Hb, RDW, SFer, sTfR, LH, α-ACT	Required to be iron depleted but not deficient Fer: 12–30 ug/L Hb <119 g/L	Monitored timing and duration of menstrual cycle and irregularities. Measured LH in each blood sample Menstrual cycle divided into phases: follicular and luteal. Luteal phase determined by estimation of ovulation based on LH levels.	No change in iron status across the menstrual cycle within iron depletion.
Angeli et al. (2016)	Ungraded	*n* = 90	19–44 years	Hb, TS, SFer, SI, SH		Measured blood samples at 6 time points. First sample collected on day 2 of menses. 3 others in menses and late follicular phase, 2 in luteal phase.	TS, SI and SH show variations throughout the menstrual cycle. SFer, Transferrin and Hb showed minimal variation but showed high degree of inter-variability TS highly correlated with SI
Zheng et al. (2021)	Silver	*n* = 8	25–45 years	Hb, Fer, Tf, SI, SH	Participant requirement for SFer to be >30 ug/L, non-deficient. Mean SFer: 59 ug/L	Measured blood samples in the early follicular (days 3–7) and mid-luteal phase (days 20–22). Repeated measures trial, samples compared here are baseline (pre-exercise) in thermoneutral conditions. Menstrual phase confirmed with retrospective serum hormonal analysis, and confirmation of ovulation with ovulatory urine test.	No difference in SH, SFer and Tf between menstrual phases throughout the trial SI appeared to be unaffected my menstrual phase.
Suzuki et al. (2018)	Ungraded	*n* = 4	19–20 years	Hb, SI, SFer, TS	1 out of 4 had SFer <35 ug/L	Blood samples collected on days 2, 10 and 22 of the menstrual cycle. Self-reported normal cycles, but no hormonal clarification	Hb and SFer peaked on day 10 of the cycle, SI and TS peaked in the luteal phase High inter- and intra-variability in participants iron status markers
Alfaro-Magallanes et al. (2022)	Silver	*n* = 36	18–40 years	SI, SFer, TS	Mean SFer for naturally menstruating females <35 ug/L, thus majority of the participant cohort would be defined as stage 1 iron deficiency	Blood samples collected in the early follicular phase (days 2–5), mid-late follicular phase (days 7–12), mid-luteal phase (days 19–24). Phase retrospectively confirmed with serum hormonal analysis	TS and SI significantly lower in early follicular vs mi-late follicular and mid-luteal phases. Iron deficiency erythropoiesis (TS <16%) greatest in early follicular phase. No association or variation in menstrua phase and iron stores

*Hb, Hemoglobin; MCV, mean corpuscular volume; SI, serum iron; TIBC, total iron binding capacity; TS, transferrin saturation; EP, erythrocytes protoporphyrin; SFer, serum ferritin; SH, Serum Hepcidin; RDW, Red cell distribution width; α-ACT, acute phase protein; sTfR, Soluble transferrin receptor; LH, Luteinizing hormone; Tf, Transferrin*.

Mean serum ferritin levels appear to remain stable across the menstrual cycle (Belza et al., 2005; Angeli et al., 2016; Lainé et al., 2016; Suzuki et al., 2018; Zheng et al., 2021; Alfaro-Magallanes et al., 2022), with some investigations reporting no change in iron parameters throughout the menstrual cycle (Belza et al., 2005; Alfaro-Magallanes et al., 2022) especially in iron-depleted individuals (serum ferritin: <12–35 μg/L). Research in athletes has demonstrated the magnitude of hepcidin activity ~3–24 h after exercise (Peeling et al., 2014) is largely influenced by their pre-exercise serum ferritin levels with minimal changes typically seen in post-exercise iron parameters and hepcidin levels in iron-deplete (serum ferritin: <35 μg/L) compared to iron replete athletes (serum ferritin >35 μg/L) (Peeling et al., 2014, 2017). Therefore, the magnitude of the changes in iron parameters and hepcidin throughout a female's menstrual cycle may also largely depend upon her current/basal serum ferritin levels.

### Acute exercise and menstrual cycle phase effect on hepcidin and iron status

Only recently have acute changes in iron parameters and hepcidin been examined before and after exercise (40 min run at 75% VO_2peak_) in three distinct phases of the menstrual cycle (early, mid-follicular phase, and luteal phase). Here, pre-exercise serum iron and IL-6 levels were lower in the early follicular phase compared to the luteal phase (Barba-Moreno et al., 2020). Despite serum hepcidin levels increasing after exercise, basal hepcidin levels showed no variation throughout the cycle (Table 2). Furthermore, neither exercise-induced changes in serum iron nor IL-6 appeared to stimulate large variations in hepcidin activity after exercise. The blunted post-exercise hepcidin response was most likely due to participants' low mean basal serum ferritin levels, ranging from 25.4 ng/mL during the early follicular phase to 29.2 ng/mL during the luteal phase, which is lower than the 35 μg/L cut off for stage 1 iron deficiency (Barba-Moreno et al., 2020). Therefore, large variations in hepcidin concentration would not be expected in this cohort since hepcidin activity has likely been suppressed to promote iron absorption in response to depleted iron stores (Galetti et al., 2021). To account for participants' low pre-exercise iron status, participants were divided into females with basal serum ferritin levels <20 and >20 μg/L and hepcidin and iron parameter responses were again reviewed before and after exercise in the early, mid-follicular, and luteal phases of the menstrual cycle. Pre-exercise hepcidin levels were significantly lower in the <20 μg/L ferritin group in the early follicular and mid-follicular phases of the cycle, but were not significantly different from the >20 μg/L ferritin group in the mid-luteal phase (Alfaro Magallanes et al., 2021a). Post-exercise, no significant difference in hepcidin levels was observed between the two groups. The results for this second study should be interpreted with caution, due to several analytical and methodological considerations. Firstly, the authors did not complete an ANOVA but rather conducted their analysis through a series of *t*-tests between the groups and time points within this study. This method may not have appropriately adjusted for the Familywise Error Rate, which could affected the statistical significance of the results they obtained (Alfaro Magallanes et al., 2021a). Secondly, basal ferritin or iron status was not included as a covariate when analyzing the effect of hepcidin activity between menstrual cycle phases or pre-and post-exercise. Therefore, the hepcidin response could have been influenced by participants' low pre-exercise iron stores, especially when considering the mean serum ferritin for the >20 μg/L was not greater than 50 μg/L, which has been suggested as a healthy serum ferritin cut-off for females (Galetti et al., 2021). Thirdly, the small sample size in each cohort (*n* = 7–8) of the studies reduces the statistical power to infer statistical differences in iron status between each menstrual cycle phase within this study.

**Table 2:** Studies that have investigated iron status response pre and post exercise at various phases in the menstrual cycle in premenopausal females.

**Author**	**Study ranking for methodological control in female cohorts (Smith et al., 2022)**	**Population characteristics and classification of level of training (McKay et al., 2022)**	**Exercise intervention**	**Phase of Cycle**	**Results on iron status and metabolism**	**Comments**
Roecker et al. (2005)	Ungraded	*n* = 14 Marathon runners 26–45 years 52–75 kg Marathon time: 3:59–4:55 No medication that would affect iron status Tier 2	Assessment of iron stores pre, post, 1 day post and 3 days post a marathon	No phase of menstrual cycle or OCP reported	Urinary hepcidin increased after marathon in 10/14 females Max hepcidin 1 day after marathon Responders (iron sufficient stores): increase in hepcidin 4–27-fold of pre-race value	No dietary control No indication of volume of training No monitoring of iron stores prior to marathon
Newlin et al. (2012)	Ungraded	*n* = 12 Female runners 19–32 years VO_2_max: 52.1 mL/kg/min Iron sufficient (Fer >30 μg/L) Tier 1	Trial 1: 60 min at 65% *v*VO_2_peak Trial 2: 120 min at 65% *v*VO_2_peak	All tests completed 7–10 days after the onset of menses. Phase likely to be late FP but no hormonal confirmation	Increase Hct and Hb post exercise Increase SH 3 h post exe for both trials, but was greater in 120 min trial SI increased post and 3 h post, lowest at 9 h post SFer increased post exercise in both trials	24 and 48 h dietary control Changes in SI and SH followed each other post exercise Responders for SH had higher SI (78.8 ug/L) vs non-responders (61.5 ug/L)
Ishibashi et al. (2017)	Ungraded	*n* = 16 Long distance runners Mean age: 20.5 years Tier 3	Blood sample collection Low training period (~499 km/month) High training period (~622 km/month)	Self-reported regular menstrual cycles in 25% in low training period vs. 19% in high training period 2 participants with amenorrhea No further clarification of menstrual phase	SFer Low: 30.9 ug/L SFer High: 28.1 ug/L SI Low: 55 ug/L SI High: 65 ug/L CK higher in high training period 31% during low were iron deficient 37% during high were iron deficient SH greater in high training period Positive correlation between SH and SFer in both training periods	0% use supplements in low training period 44% used supplements in high training period
Barba-Moreno et al. (2020)	Bronze	*n* = 15 Female athletes VO_2_max:50.3 mL/kg/min Mean age: 35.6 years Tier 1	Baseline testing in early FP (days 2–5 of cycle), followed by 3 trials • Early FP: days 2–5 and low hormone • Mid FP days 7–10, high E2 and low P4 • LP days 19–21, high P4 and E2 5 min warm up at 60% vVO_2_, 40 min running at 75% of vVO_2_peak, 5 min recovery at 30% vVO_2_peak.	Natural cycles, occurring between 24–24 days in length Blood sample taken upon arrival at the lab prior to exercise and retrospectively analzsed for E2, P4, LH and FSH. No LH surge to confirm ovulation Deficient luteal phase defined as P4 <16 nmol/L in single measurement	Effect of time differences found for transferrin, SFer, IL-6 and SH. Significant main effects were found for SI and menstrual cycle, while CRP and IL-6 showed a trend toward significance. No significant changes in SH, SFer and transferrin throughout the menstrual cycle.	Only significant interaction effect for menstrual cycle and time was for IL-6. With an increase from pre to 0 h and further to 3 h post-exercise. Increase significantly greater in LP vs Early FP and Mid FP. Peak in SH at 0 h post exe vs. 3 h post.

*BM, Body mass; VO_2peak_, Maximal aerobic capacity; vVO_2peak_, Velocity at maximal aerobic capacity; Hb, Hemoglobin; Hct, Haematocrit; SI, Serum iron; SH, Serum Hepcidin; SFer, Serum ferritin; TS, Transferrin Saturation; CK, Creatine kinase; IL-6, Interleukin-6; OCP, Oral contraceptive pill; FP, Follicular phase; LP, Luteal phase; MHR, Maximal heart rate; LH, Luteinizing hormone; FSH, Follicle stimulating hormone; E2, estrogen; P4, Progesterone; CRP, C-reactive protein; BMI, Body mass index*.

Another potential limitation of the previous two studies is their use of microplate ELISA (Elabscience Human Hep25 ELISA kit) to measure hepcidin, which is limited compared to the mass spectrometry method. ELISAs may not identify the active, full length of the 25-amino acid hormone from truncated variants that are present due to biological breakdown that occurs during sample collection and sample analysis (Hare, 2017). Hepcidin measured *via* matrix-assisted laser desorption ionization weak cation exchange time of flight (MALDI-TOF) mass spectroscopy is currently considered the gold standard measurement method. A round-robin investigation on hepcidin analysis (mass spectrometry and ELISA's) showed significant variation in hepcidin levels between eight different laboratories for a single analysis (Kroot et al., 2009). Several competitive and sandwich-based ELISA's for hepcidin analysis are available, however, when compared to mass spectrometry the results obtained by ELISA's may lack the sensitivity of detecting hepcidin in biological fluids (i.e., plasma, serum, or urine) (Kroot et al., 2009; Hare, 2017). For example, in male and female athletes who presented with iron deficiency, their mean serum ferritin of ~20 μg/L resulted in suppressed mean serum hepcidin of 20–30 ng/mL (Burden et al., 2015). Following treatment with IV iron (500 mg Ferinject; Vifor Pharma Ltd., Opfikon, Switzerland), serum ferritin has been shown to increase to ~100–120 μg/L, with subsequent increases in serum hepcidin to ~100–150 ng/mL (Burden et al., 2015). In the aforementioned studies, the eumenorrheic female participants had mean serum ferritin levels of 25.4 ng/mL, yet their hepcidin levels ranged from 60 to 80 ng/mL (Barba-Moreno et al., 2020). The serum hepcidin levels reported by Barba-Moreno et al. (2020) and Alfaro Magallanes et al. (2021a), appear to be inconsistent with prior research in iron depleted individuals. Rather, the reported hepcidin levels appear equivalent to those measured in athletes after IV iron treatment (Burden et al., 2015). Such discrepancies in the results may be due to the variation in hepcidin ELISA measurements, therefore we recommend thorough reviews of hepcidin ELISA values against mass spectroscopy and existing literature to ensure consistency in results published within this research area.

Few studies have investigated changes in iron status in active females over a prolonged period (weeks-months) (Table 3). None of the available research has considered monitoring menstrual cycle status while recording changes in iron status in the female participants. This prior research lacks consideration of confounding variables in females that may affect iron status. Hence, we encourage researchers looking to complete prolonged investigations of iron status in females to ensure menstrual cycle length, bleeding, and symptoms are monitored to allow a more nuanced analysis.

**Table 3:** Studies that have investigated changes in hepcidin and iron parameters pre and post chronic training periods in premenopausal females.

**Author**	**Study ranking for methodological control in female cohorts (Smith et al., 2022)**	**Population characteristics and classification of level of training (McKay et al., 2022)**	**Exercise intervention**	**Phase of Cycle**	**Results on iron status and metabolism**	**Comments**
Auersperger et al. (2013)	Ungraded	*n* = 14 Female runners Divided into 2 groups N: Fer>20 μg/L D: Fer <20 μg/L Tier 1	2-week prep phase 8-week intensified training 2 ×3-week progressive overload periods followed by 1 week taper 3–4 sessions per week 1–2 interval sessions at 88–100% MHR) 2 aerobic runs per week (70–85% MHR)	No recording of menstrual cycle or changes	SH decreased in recovery weeks vs baseline Prevalence of Fer <20 μg/L increased to 71% (10/14) in 8 weeks	N group had a 4.8% improvement in performance D group had a 3% improvement in performance IL-6 in detectable ranges at all time points No significant group effects for CRP
Ma et al. (2013)	Ungraded	*n* = 20 Female runners Control n=10 age and BMI matched females Age 18–23 years Tier 4	Weekly running volume in runners ranged from 56.3–104.6 km Average run volume 24 h prior to the blood test in runners was 10.5 km Run pre-blood test was continuous, moderate effort (~12.9–13.7 kph)	Blood collections between 15 and 19th day of cycle. Phase likely to be early LP. No hormonal confirmation of reproductive hormones or LH for ovulation	SFer tended to be higher in runners SI tended to be lower in runners SH tended to be higher in runners	Regular menstrual cycles reported in 80% controls and 70% runners Exercise was completed prior to the morning sample. Run length ranged from 0 to 19.3 km, likely to contribute to the high SH and SFer in the runners
Buyukyazi et al. (2017)	Ungraded	*N* = 30 *N* = 8 control group and no exercise Age: 43.5 years* VO_2_peak: 23.9 mL/kg/min* *N* = 22 experimental group BWG =11 brisk walking group Age: 41.0 years* VO_2_peak:34.1 mL/kg/min* MWG = 11 moderate tempo walking group Age: 38.0 VO_2_peak:31.4 mL/kg/min* Not taking iron supplements, regular length menstrual cycle for 12 months and not taking any other medication Tier 0	Week 1: low intensity for all to gain familiarity with protocols 8-week walking intervention for 2 groups at 2 different intensities. Start at 30 min and build to 40 min in duration in first 4 weeks. 3 × a week on a 400 m track BWG: weeks 1–4 70% MHR, weeks 5–8: 75% MHR MWG: weeks 1–4 50% MHR, weeks 5–8: 55% MHR Intensity determined by Karvonen Equation	No recording of menstrual cycle	Baseline iron status: BWG: 10.3 ng/mL* MWG: 10.7 ng/mL* CG:10.7 ng/mL* SH increased post exercise intervention in BWG, MWG and CG. IL-6 decreased in the BWG and MWG. No change SI TIBC increased BWG Increase in Hct and RBC in BWG No change SFer, TS	Baseline iron parameters (serum ferritin and iron) were not statistically different between groups Morning rested blood collection pre and post exercise intervention. Bloods collected ~24-h post and VO_2_peak ~48 h post No screening of iron status Measured pro-hepcidin a prohormone form. Minimal comparisons to gold standard and other studies in exercise-based studies. ~Stage 2 iron deficiency, degree of change expected in 8 weeks is minimal. Hepcidin levels likely to be low in iron depleted individuals.

BM, Body mass; VO_2peak_, Maximal aerobic capacity; vVO_2peak_, Velocity at maximal aerobic capacity; Hb, Hemoglobin; Hct, Haematocrit; SI, Serum iron; SH, Serum Hepcidin; SFer, Serum ferritin; TS, Transferrin Saturation; CK, Creatine kinase; IL-6, Interleukin-6; OCP, Oral contraceptive pill; FP, Follicular phase; LP, Luteal phase; MHR, Maximal heart rate; LH, Luteinizing hormone; FSH, Follicle stimulating hormone; E2, estrogen; P4, Progesterone; CRP, C-reactive protein; BMI, Body mass index; BWG, Brisk walking group; MWG, Moderate tempo walking group. *Median presented in report and in table.

### The effects of acute exercise and oral contraceptive pill phase on hepcidin and iron status

The oral contraceptive pill (OCP) is a widely and commonly used contraceptive method for menstruating females. In a cohort of 430 female athletes, ~50% used a form of hormonal contraception, of which ~68% used an OCP (Martin et al., 2018). Forms of OCP include, the combined monophasic pill providing a low dose of both ethinylestradiol and synthetic progestin (levonorgestrel, norethisterone, gestodene, desogestrel, drospirenone) consistently for 21 days, the biphasic pill providing consistent doses of ethinylestradiol and higher doses of synthetic progestins in the second half of the active pill cycle and the triphasic pill with synthetic ethinylestradiol and progestin doses changing every 7 days during the active pill phase. Typically, most OCPs will have a 21-day active or consumption phase and a 7-day pill-free/withdrawal phase (Elliott-Sale et al., 2020). Doses of the exogenous E2 and progestins within combined OCPs may range from ~20 to 40 mg of ethinylestradiol and ~70–300 mg of synthetic progestins (Elliott-Sale et al., 2013). An alternative to the combined OCP is the progesterone-only pill, providing doses of ~35–75 mg of synthetic progestins (Elliott-Sale et al., 2013). The provision of exogenous E2 and progestins have a negative feedback on gonadotrophic hormones, prolonging the downregulation of the hypothalamic-pituitary-ovarian axis, subsequently resulting in significant reductions of endogenous E2 (~60 pmol/L for 21 days) and P4 (consistently ~5 nmol/L) within premenopausal females (Rechichi et al., 2008; Elliott-Sale et al., 2020; Alfaro-Magallanes et al., 2022). One hour following daily ingestion of the OCP, synthetic E2, and P4 peak, and during the 21-day active pill period the basal values of the synthetic hormones may slightly increase (Elliott-Sale et al., 2020). During the seven pill-free or placebo pill days, endogenous E2 levels may rise to ~140 pmol/L, equivalent to E2 levels in the early follicular phase (Elliott-Sale et al., 2020).

In general, the magnitude of the hepcidin response following exercise does not seem to differ between OCP users and non-users (Table 4). Two studies have investigated the hepcidin response pre-and post-exercise in female athletes during the withdrawal and active pill phases. Both research studies showed the typical increase in hepcidin post-exercise, however, no significant difference between the active and withdrawal trials on hepcidin activity pre-and post-exercise was observed. The low sample sizes in the research investigating the influence of the OCP on iron metabolism makes it difficult to draw clear conclusions (Sim et al., 2015, 2017; Alfaro-Magallanes et al., 2021b). Aligning with previous research, hepcidin kinetics are likely to have responded to participants' iron status, with higher hepcidin levels reported in the participants of Sim et al. (2015) (43–47 μg/L) who had higher iron stores, and inflammatory increases post-exercise rather than steroid sex hormone fluctuations associated with OCP use. Neither of these studies included a control group (non-OCP, basal iron status matched) to compare the post-exercise hepcidin response between OCP users and non-users. Additionally, while both research investigations tried to capture a hormone-free period and an active hormone phase, only one study (Alfaro-Magallanes et al., 2021b) attempted to account for the increase in endogenous E2 in the withdrawal phase, while one did not (Sim et al., 2015), which may have influenced hepcidin kinetics and iron parameters during this data collection period.

**Table 4:** Studies that have investigated basal iron status and pre to post exercise iron status changes during the withdrawal and active phases of the oral contraceptive pill (OCP).

**Author**	**Study ranking for methodological control in female cohorts (Smith et al., 2022)**	**Population characteristics and classification of level of training (McKay et al., 2022)**	**Exercise intervention**	**Phase of OCP**	**Results on iron status and metabolism**	**Comments**
Sim et al. (2015)	Silver	*n* = 10 Mean age 26 years Mean BM: 57 kg Mean VO_2_max: 50.3 mL/kg/min Tier 1	40 min run at 75% vVO_2_peak Withdrawal: Day 2–4: hormone free period Active: Day 12–14: end of week 1 of active hormone therapy	On OCP for a minimum of 3 months. 3 brands: 1. Leven 2. Yasmin 3. Estelle OCP ethinyl estradiol ranges 0.03–0.035 mg OCP progestogen ranges 0.15–3 mg	SI increased immediately post exercise and remained elevated 3 h post in withdrawal trial. SFer increased post exercise in both trials and remained high 3 h post in withdrawal, only trending to remain high in active trial. Increase in SH in both trials with no difference between trials. No correlation between IL-6 and SH Moderate relationship between SH and SFer in active trial SI and SH not related 3 h post exercise	Time course of hormones post ingestion or cumulative levels after 1 week of active pill ingestion not quantified All sessions completed in the morning, when there is likely to be a spike in exogenous hormones Testing in withdrawal phase likely to occur at a time point when there is a rebound in endogenous estrogen equivalent to early FP
Sim et al. (2017)	Silver	*n* = 15 Mean age: 25 years Mean BM: 56.4 kg Tier 1	-	On OCP for a minimum of 3 months. 3 brands: 1. Leven 2. Yasmin 3. Estelle OCP ethinyl estradiol ranges 0.03–0.035 mg OCP progestogen ranges 0.15–3 mg	No difference in SI, SH, TS between withdrawal and active pill phases Serum ferritin significantly higher in active pill phase compared to withdrawal phase (69.4 vs 61.1 ug/L respectively)	Time course of hormones post ingestion or cumulative levels after 1 week of active pill ingestion not quantified All sessions completed in the morning, when there is likely to be a spike in exogenous hormones Testing in withdrawal phase likely to occur at a time point when there is a rebound in endogenous estrogen equivalent to early FP
Alfaro-Magallanes et al. (2021b)	Silver	*n* = 16 Female athletes: VO_2_peak 47.4 mL/kg/min Mean age: 25.3 years Not iron deficient Tier 1	3 visits over 2 OCP phases. 1. Baseline in withdrawal phase 2. Trial in withdrawal phase (~day 4.9) 3. Trial in active pill phase (~ day 22.1) Exercise task: 5 min warm-up at 60% *v*VO_2_peak, 8 x 3 min at 85% vVO_2peak_, with 90 s recovery at 30% *v*VO_2_peak. 5 min cool down at 30% vVO_2_peak Serum samples at pre- exercise, 0 h, 3 h post and 24 h post exercise. Completed between 8 and 10 a.m.	All previously used OCP for 6 months prior Different formulas of OCP used and not controlled for. OCP ethinylestradiol ranges 0.02–35 mg OCP progestogen ranges 0.075–250 mg OCP use for study: 7 days (Days 1–7) no hormonal load/withdrawal phase 21 days of exogenous hormone pills/active phase	No significant difference in SH, IL-6, CRP and SFer between OCP phases No significant differences between the OCP phases at any time point pre and post exercise for SH and SFer Trend for hepcidin to be higher at baseline in withdrawal phase Trend for CRP to be higher in the withdrawal phase TNF-α significantly higher in active phase SI significantly higher at all time points in active pill phase Transferrin significantly lower in active phase at 0, 3, and 24 h post exercise.,	17β-estradiol, LH and FSH were significantly lower in active pill phase vs withdrawal phase Time of SH measurement affected results. SH was significantly higher at 0 h and 3 h post-exercise vs pre-exercise in withdrawal phase. IL-6 was significantly higher at 0 h, 3 h and 24 h post-exercise in both phases. SFer higher levels 0 h post-exercise vs preexercise in both withdrawal and active phases. TNF-α higher 0 h post-exercise vs pre-exercise in withdrawal phase Transferrin significantly higher 0 h post exercise vs pre-exercise and 24 h post exercise in active phase No note of when the OCP was ingested by participants and if this may have influenced the results
Alfaro-Magallanes et al. (2022)	Silver	n=24 Mean age: 27 years Mean BM: 57.25 kgs VO_2_peak 49.4 mL/kg/min Tier 1	-	All previously used OCP for 6 months prior Different formulas of OCP used and not controlled for. Pill ingested every night for 21 days OCP ethinylestradiol ranges 0.02–0.03 mg OCP progestogen ranges 0.01–3 mg Tested late withdrawal phase ~day 5 to coincide endogenous estrogen peak Active pill phase tested on ~day 20 to coincide reduced ovarian functioning and peak in exogenous hormone concentrations	Ts and SI significantly higher in the active pill phase. Tf was lower in the active pill phase	Samples collected in rested and fasted state Endogenous 17β-estradiol in withdrawal and active pill phases were 31.51 and 9.81 pg/ml, respectively Endogenous progesterone in withdrawal and active pill phases were 0.37 and 0.36 ng/ml respectively Blood samples were collected in the morning (8–10 a.m.) to reduce the influence of diurnal variation. Timing was greater than 6 h and potentially coincided with reduced levels due to metabolic breakdown following ingestion.

*BM, Body mass; VO_2peak_, Maximal aerobic capacity; vVO_2peak_, Velocity at maximal aerobic capacity; Hb, Hemoglobin; Hct, Haematocrit; SI, Serum iron; SH, Serum Hepcidin; SFer, Serum ferritin; TS, Transferrin Saturation; Tf, Transferrin; CK, Creatine kinase; IL-6, Interleukin-6; OCP, Oral contraceptive pill; FP, Follicular phase; LP, Luteal phase; MHR, Maximal heart rate; LH, Luteinizing hormone; FSH, Follicle stimulating hormone; E2, estrogen; P4, Progesterone; CRP, C-reactive protein; BMI, Body mass index*.

More recently, basal iron status has been compared between three phases of the menstrual cycle in eumenorrheic females, with the withdrawal and active pill phases of females on the OCP (Alfaro-Magallanes et al., 2022). Regardless of the menstrual cycle or OCP phase, serum ferritin, hemoglobin and IL-6 did not display any variations between phases (Alfaro-Magallanes et al., 2022), consistent with research suggesting there is no difference in the proportion of menstruating females and females on the OCP who presented with iron deficiency (Casabellata et al., 2007). The average serum ferritin for this study appears to have been ~30–35 μg/L, which we have previously noted may limit the magnitude of change in iron parameters between phases. Lower transferrin saturation, iron, and transferrin were noted in the early follicular phase in eumenorrheic females when compared to the withdrawal and active pill phases. In addition, these same iron parameters were lower in the mid-late follicular phase and mid-luteal phase when compared to the active pill phase. Elevated transferrin and serum iron (~1.2 fold higher) have been reported in OCP females, from the first to the third generation of OCP pills (Casabellata et al., 2007), with females on these OCPs more likely to have transferrin saturation levels >45% and be considered at risk of hemochromatosis (McKnight et al., 1980). These results are likely due to the hepatic effects of synthetic estrogens (McKnight et al., 1980), offering a potential explanation for the difference in iron parameters noted between eumenorrheic and OCP users (Alfaro-Magallanes et al., 2022). Therefore, the influence of variations in synthetic estrogens and progestin in the first through to the third generation of OCPs on iron parameters will need to be verified, as these synthetic sex hormones may be confounding our interpretation of iron status in OCP users (Casabellata et al., 2007).

Interestingly, different brands of the monophasic pill result in different levels of endogenous hormones. Research has demonstrated that 11 out of 30 OCP brands tested (~37%) will produce significantly different levels of endogenous E2 and P4 in both the active and withdrawal phases (Elliott-Sale et al., 2013). The different E2 and P4 hormone concentrations between women may arise due to different doses of exogenous E2 and P4 in each pill (brand and type), differential metabolism of exogenous hormones which is influenced by an individual's genetics, and the subsequent effects of these different endogenous hormone levels on hypothalamic-pituitary-gonadal feedback loops, and their interactions with catecholamines and prostaglandins (Yen, 1977; Elliott-Sale et al., 2013). Without controlling the OCP brand used by participants in research studies, there will likely be large variations in endogenous hormone levels, which may then be summated to calculate the mean differences between OCP users and non-users. This raises several methodological concerns (Type II errors) for the accurate interpretation of how changes in endogenous hormone concentrations in OCP users affect iron parameters and hepcidin activity.

Behavioral OCP use including the timing of OCP ingestion (morning or evening and peak of synthetic hormones post-ingestion) relative to the measurement of iron parameters will need to be considered in study designs and when interpreting iron status in athletic females, given reproductive hormones can affect iron parameters. Additionally, considerations for athletic females to avoid the 7-day withdrawal pills in favor of continuation of the active pills to reduce bleeding incidence and manipulation of their cycle length (Schaumberg et al., 2018), have not been considered in prior research and may contribute to the lower prevalence of iron depletion in these females. Therefore, researchers and practitioners should take note of the method of OCP use in females and how this may influence the iron parameters that are being assessed.

A final consideration for both researchers and practitioners is that no research to date has investigated the effect of the progestin-only pill or other forms of hormonal contraceptives (hormonal IUD, Depo Provera injection) on hepcidin kinetics. The synthetic dose of progestin is in some cases equivalent to the P4 doses provided daily in IVF treatment. Women receiving P4 during IVF have significantly increased hepcidin levels after 6–15 days (Li et al., 2015), therefore a similar hepcidin response may be observed in iron sufficient females on the progestin only pill.

## Changes to the diagnosis of iron depletion in premenopausal females

Recent reviews have set out a framework for regular iron status monitoring of athletes (Sim et al., 2019), however, there is large variability in the optimal ferritin levels that have been suggested. Whilst stage 1 iron deficiency has been defined as serum ferritin <35 μg/L (Peeling et al., 2014), some authors advocate for a higher serum ferritin cut offs, such as 50 μg/L, for performance, health, and to aid adaptation to training loads (Peeling et al., 2014; Galetti et al., 2021). This elevated serum ferritin threshold is based on sustained increases in hepcidin following exercise (1–3 sessions per day), in conjunction with exercise-induced iron loss mechanisms each session increasing the risk of developing a negative iron balance (Fensham et al., 2022). Subsequently this then increases the risk of being diagnosed with an iron deficiency and experiencing the adverse health and performance outcomes associated with this diagnosis.

Recent research has provided physiological support for a higher serum ferritin threshold for the diagnosis of iron deficiency based on the up-regulation of iron absorption and recycling (Galetti et al., 2021). Results from this investigation demonstrated an exponential relationship between serum ferritin and hepcidin, with 41.8% of the variation in fractional iron absorption in healthy premenopausal females explained by variations in hepcidin. Key results indicated that fractional iron absorption increased from 7.2% to 33.2% when serum hepcidin was ≤ 3.09 nmol/L. The corresponding serum ferritin was ~51.1 μg/L, and similarly, fractional iron absorption increased when serum ferritin concentration was <51.1 μg/L but remained relatively stable when hepcidin and serum ferritin levels were >3.09 nmol/L and >51.1 μg/L, respectively. Raising the serum ferritin threshold for iron depletion to ~50 μg/L in active females may increase the sensitivity in detecting iron depletion. The benefit of early detection of low iron levels affords early correction through dietary means and oral iron supplementation if required (for a review of iron treatment strategies see McCormick et al., 2020).

The wide confidence intervals for both hepcidin and serum ferritin in the study by Galetti et al. (2021) represent the large inter-individual variability for both serum ferritin and hepcidin measurements that exists within the females. While population-derived serum ferritin cut-offs provide some information about an individual's iron status and risk of iron depletion/deficiency, regular medical and iron status screening is still strongly advised for active females to determine the typical intra-individual variation in hematological and iron parameters throughout the competitive season or sporting career.

## Heavy menstrual bleeding in premenopausal females-implications and considerations for iron status

Sequential exposure of the E2 primed endometrium to P4 and subsequent withdrawal of P4 and E2 in the late luteal phase initiates spontaneous decidualization of the endometrium resulting in menstrual bleeding (Hapangama and Bulmer, 2016). Mean menstrual blood loss in premenopausal females may range from 6.55 to 178.7 mL, with ~63% of women losing between 1 and 40 mL of menstrual blood (Hallberg et al., 1966). Many females with normal menstrual blood loss (1–40 mL), may present with a healthy and normal iron status (Hb >12 g/100 mL, serum iron >104 μg/100 mL; Hallberg et al., 1966), suggesting that normal menstruation may not be a root cause of iron deficiency in premenopausal eumenorrheic females.

However, endocrine irregularities (structural and non-structural) that prevent these sequential events have the potential to result in heavy menstrual bleeding (HMB) (Hapangama and Bulmer, 2016). Heavy menstrual (or uterine) bleeding, formerly called menorrhagia, is defined as a total menstrual fluid loss that regularly exceeds 80 mL (Hapangama and Bulmer, 2016). Females who present with HMB may have iron losses that are approximately 5–6 times higher during menstruation, with >80 mL of menstrual fluid loss equating to ~5.2 μg/L of iron loss (Napolitano et al., 2014). As such, HMB has consistently been suggested to increase the risk of iron deficiency (Hallberg et al., 1966; Napolitano et al., 2014; Bruinvels et al., 2016). A handful of research has attempted to quantify the incidence of HMB in sedentary females (Hallberg et al., 1966; Napolitano et al., 2014) and active females (Bruinvels et al., 2016), however, most of this research has based its results on the participants' subjective (survey) opinion or perception of their menstrual bleeding volume (Fraser et al., 2001; Bruinvels et al., 2016). There is no validated survey for the detection of HMB, and many females are unable to accurately estimate the volume of blood lost during menstruation, and appear to have low awareness of what constitutes as a healthy/normal compared to a heavy volume of menstrual blood lost during menstruation (Hallberg et al., 1966; Mansour et al., 2021). In a cohort of females classified with HMB (>80 mL), 37% considered this to be only moderate menstrual blood loss, while 4% considered this scant menstrual blood loss. In contrast, 14% of females that presented with 20 mL of menstrual blood loss considered this to be HMB (Hallberg et al., 1966). Given the current lack of validated HMB measures, the prevalence of HMB within athletic premenopausal females may have been underestimated.

The relationship between iron deficiency and HMB prevalence has been, to date, limited to premenopausal females with a healthy cycle (Bruinvels et al., 2016; Mansour et al., 2021). Minimal consideration has been given to how variations in menstrual cycle status (non-structural cause/s of HMB), and reproductive hormone profiles affect bleeding characteristics (volume, duration). Alterations to menstrual bleeding patterns/characteristics may occur in response to the adaption of the hypothalamic-pituitary-ovarian axis, typically presenting as ovarian suppression and resulting in changes in reproductive hormone concentration with subsequent effects on endometrial proliferation (Brown and Thomas, 2011). Changes to cycle length (oligomenorrhea), cycle characteristics (HMB) and sex hormone levels (subclinical ovulatory disturbances e.g., luteal phase defects or anovulation) can occur at any age in healthy premenopausal women in response to physiological [regular exercise (Shangold et al., 1979; De Souza et al., 1998; De Souza, 2003); or altered dietary intake (Bedford and Barr, 2010)] and psychological stressors (Barr, 1999).

Initial changes to menstrual cycle status may manifest as a subclinical ovulatory disturbances (SOD) inclusive of luteal phase defects (LPD), which may present as short luteal phases (<10 days) or inadequate/low P4 levels (<16 nmol/L), or anovulation (failure to ovulate and subsequently low P4 levels, <16 nmol/L). A SOD cycle in healthy premenopausal females typically remains undiagnosed as the menstrual cycle length (number of days) does not change (De Souza, 2003). However, the changes in reproductive hormone concentrations within SOD cycles, specifically E2 excess and low/lack of P4 are associated with compromised differentiation of the endometrial lining, resulting in poor quality of the endometrium and infertility in that cycle (De Souza, 2003). The role of P4 in the endometrium is to reduce the biologic activity of E2, transitioning the endometrium from the proliferation phase to the secretory phase, with menstrual bleeding then initiated by P4 withdrawal. However, in the presence of low P4 (e.g., LDP or anovulatory cycle), the reduction of E2-induced proliferation is likely to be low, resulting in excessive endometrial growth or delayed initiation of menstrual bleeding. Therefore, during the early follicular phase, following the withdrawal of E2 and P4 and spontaneous decidualization of the endometrial tissue, females presenting with LPD or anovulatory cycles, may have altered menstrual bleeding patterns, possibly including either HMB or prolonged menstrual bleeding ≥7 days. Evidence to support the relationship between endocrine irregularities and HMB prevalence has been reported in obese females (but not diagnosed with PCOS) and females diagnosed with PCOS (who may or may not be obese) (Hapangama and Bulmer, 2016). In females diagnosed with PCOS (obese and not obese), excess androgens are converted to estrone in peripheral tissues, with prolonged periods of excessive and unopposed E2 action on the endometrium resulting in HMB (Hapangama and Bulmer, 2016). In obese females, with no current PCOS diagnosis, the conversion of androstenedione secreted by the adrenal glands to estrone in adipose tissue provides an additional source of E2 that supports excessive endometrial growth and HMB (Hapangama and Bulmer, 2016).

The prevalence of SOD cycles in healthy females with normal length menstrual cycles (21–35 days) in 2 and 6 month prospective studies was 16 and 22.9% respectively (Bedford et al., 2010; Schliep et al., 2014). Within active females, the prevalence of anovulatory and LPD cycles are relatively common, with a 48 and 79% prevalence respectively in a 3-month study (De Souza et al., 1998). Within this study, ~46% of females had consistent length cycles but had inconsistent cycle-to-cycle hormonal variations with intermittent presentations of ovulatory, anovulatory, and LPD cycles. Approximately 33% of the recreationally active females presented with consistent LPD cycles (De Souza et al., 1998). In a cohort of 120 healthy non-active females, 16% experienced HMB that was caused by a SOD cycle (Khan et al., 2016). Self-reported HMB in 54 and 36% in active females (*n* = 789) and marathon runners (*n* = 1,073), respectively have been reported in survey-based data (Bruinvels et al., 2016). The prevalence of previous iron deficiency in those who reported HMB was 43.1 and 38.1% in active females and marathon runners, respectively (Bruinvels et al., 2016). A limitation of this study was that no time frame was set for when participants may have experienced HMB before the marathon or data collection. Therefore, we are unable to determine if HMB occurred closer to the marathon when exercise and psychological stress may have been high and may have increased the likelihood of a SOD cycle occurring. Additionally, the age or other characteristics/factors (other menstrual disorders e.g., PCOS, body mass, nutritional intake, past pregnancy) of the active females or marathon runners were not presented. Regardless, the high prevalence of SOD cycles and association with HMB in active females may provide some insight into why active females may be more prone to iron deficiency than their sedentary counterparts.

Historically OCPs have been used to treat HMB and may reduce menstrual bleeding by ~50% (Larsson et al., 1992; Mansour et al., 2021). The suppressed endogenous E2 and P4 levels that occur with OCP use are known to result in atrophy of the endometrium, poorly developed or absent spiral arterioles in the endometrium, and affect coagulation, fibrinolysis and prostaglandin synthesis (Larsson et al., 1992). These changes help to minimize menstrual blood loss in OCP users and reduce the risk of iron deficiency in these females. Thus, variations in endogenous E2 and P4 production, either suppressed by OCPs, or high E2 and low P4 in LPD and anovulation cycles in naturally menstruating females may affect menstrual bleeding patterns in premenopausal females and the relative risk of iron deficiency.

Altered hypothalamic-pituitary-ovarian axis and sex hormones function has been widely considered in the clinical sequelae of Relative Energy Deficiency in Sport (RED-S) syndrome in female athletes (Mountjoy et al., 2014; Williams et al., 2019). Research has consistently shown a reduction in triiodothyronine (T_3_), within athletes presenting with RED-S symptoms, a result that is proposed to occur due to the adaption of the hypothalamic-pituitary-thyroid axis to reduced energy intake (McCall and Ackerman, 2019). Changes in circulating T_3_ appear to be correlated with both the induction and reversal of ovulatory disturbances in females (Williams et al., 2001). The suppression of the hypothalamic-pituitary-ovarian axis during a hypometabolic state has been significantly associated with T_3_ levels and not weight loss in monkeys (Williams et al., 2001). Therefore, restoring energy balance, either by reducing energy expenditure or increasing energy intake, may provide a sufficient stimulus to reverse the hypometabolic adaptation of the hypothalamic-pituitary-ovarian axis, aiding the recovery of a fully eumenorrheic cycle. Endometrium quality and thickness have also been correlated with energy availability, reproductive hormonal environment, and iron status, such that females with optimal iron status and ovulatory function present with both increased endometrial thickness and quality (Clancy et al., 2006). During intense training periods, when the incidence of HMB may increase in addition to exercise accelerating iron loss, female athletes should be encouraged to regularly check their iron status, ensure they are ingesting sufficient fuel to support training and health (achieving optimal energy availability) and track their menstrual cycle length, bleeding length and intensity of blood loss (e.g., by using smartphone application/s).

## Practical recommendations to maintain iron status in premenopausal females

The following practical recommendations are aimed at improving iron status in active, premenopausal females:

1. Consider raising the serum ferritin threshold to 50 μg/L.
2. Ensure screening every 2–3 months for individuals considered at risk of iron deficiency, including athletes, vegans, vegetarians, and individuals traveling to or training at altitude).
3. Consider food first interventions with an accredited Sports Dietitian, providing nutritional interventions/support aimed at increasing the overall amount of iron within the diet and adequate energy and carbohydrate availability. However, iron supplements should be provided and are recommended when required (e.g., if iron depleted or deficient).
4. Regular and consistent tracking of the menstrual cycle for all menstruating females is recommended, specifically cycle length, menstrual bleeding length, relative heaviness of a menstrual bleed, and change or prevalence of premenstrual symptoms (de Paula Oliveira et al., 2021).
  - For simplicity, we suggest athletes use smartphone-based applications, currently there are several commercial options available to athletes, recreationally active individuals, and coaches that can be used for this purpose (e.g., Clue™, WILD AI™, FitrWoman™, and Garmin™).
5. Education of athletes, coaches, and sport science support staff on the menstrual cycle, cycle to cycle variations, menstrual cycle dysfunctions, and the burden of iron deficiency on health and exercise performance and training ability.
  - This may include nutritional education, psychological support (e.g., information about managing pressures on body image, competition pressure, work, and life stress).
6. We recommend that the individual's phase of the menstrual cycle, and any hormonal (OCP type and brand, hormonal IUD) or non-hormonal contraceptive (copper IUD) be recorded when performing hematological screening to enable the correct determination of iron status.

### Directions and recommendations for future research

Future research may consider the following to help improve our understanding of iron regulation in premenopausal females.

1. In naturally menstruating females, research should seek to clarify the *in vivo* influence of sex steroid hormones, inflammatory cytokines, and systemic iron levels on hepcidin throughout a eumenorrheic cycle. To date, there is minimal research on females that would allow us to comprehensively state the effects of endogenous hormones in the menstrual cycle on iron status. More research of this nature is required in iron sufficient individuals.
2. Seek to clarify if increased inflammation in the late luteal phase, aligning with the initiation with menstrual bleeding is associated with an inflammatory-driven increase in hepcidin prior to the decrease that occurs during menstrual bleeding (early follicular) phase.
3. An examination of the interactions between the severity of PMS, inflammatory cytokine levels, and iron status may better establish the role of iron status in premenopausal females exhibiting PMS.
4. Researchers should consider the female menstrual cycle status before and during study participation and consider including females that present with ovulatory disturbances (LPD or anovulatory cycles) within research trials. This research may provide insight into the risk of iron deficiency with changes in sex steroid hormone concentration and menstrual bleeding characteristics. To date, iron regulation has been predominantly investigated in normally menstruating or eumenorrheic females. Any female participants presenting with ovulatory (e.g., luteal phase defects, anovulation) and cycle disturbances (e.g., oligomenorrhea, and secondary amenorrhea) that result in differing concentrations of sex hormones within their menstrual cycle, or changes to menstrual cycle bleeding (i.e., heavier, or prolonged bleeding) characteristics, have either been removed from a data set or have not been identified in research.
5. Few studies have investigated the changes in iron status in active females while also considering their menstrual cycle over a prolonged training period. According to recent guidelines, almost all the existing research conducted on active females is of a low quality (Table 3). This is an area that requires additional research to enhance our understanding of the changes in iron status over a prolonged training period. Such research may benefit our understanding of if there is an increase in the incidence of LPD or anovulatory cycles and whether this then exacerbates the negative iron balance and risk of iron deficiency. The recent reviews by Elliott-Sale et al. (2021) and Smith et al. (2022) provide a good discussion of how to improve the quality of research conducted with female athletes.
6. Female athletes are considered an at-risk population for low energy availability, RED-S, HMB, and iron deficiency. Research may seek to clarify any interrelationship between these conditions based on the evidence presented within this review.
7. With only four research trials to date investigating the effect of OCP use on iron status in active females, two during exercise and two throughout the respective phases of OCP ingestion, more research is required to determine whether the provision of exogenous reproductive hormones has an impact on iron status and regulation in premenopausal females. Considerations for these studies include; comparisons to iron status matched naturally menstruating females (control group), iron status in respective phases (i.e., withdrawal phase and menses or active pill and mid-luteal) in iron replete females, the brand and dose of exogenous hormones provided, the impact of progestin-only OCP on iron status, and the impact of other hormonal contraceptives (e.g., hormonal IUD, Depo Provera injection, contraceptive implant), prior behavioral use of OCP (skipped placebo pills) on iron status and hepcidin.
8. Future research may also need to give some consideration to genetic variability in hepatic cytochrome p450 (CYPs), which is responsible for the heterogeneity in OCP metabolism observed between females (Lynch and Price, 2007). It is probable that inter-individual differences in CYP expression affects the rate of metabolism of exogenous hormones from the OCP, impacting the rate of disappearance from the individual's system and possibly the subsequent effects that the OCP may have on iron regulation. To date, genetic variability in OCP metabolism and exogenous hormones has not been considered in research and may require future investigations.
9. Additionally, exogenous E2 induces CYP expression (Zhang et al., 2018). Future research may seek to investigate the impact of varying doses of E2 in the OCP and the genetic expression of CYP in females on iron regulation.
10. Research is required to update HMB prevalence rates using validated subjective tools in healthy premenopausal females. Validated menstrual blood loss tools, such as the menstrual pictogram (Magnay et al., 2014, 2018), may be used to educate premenopausal females on HMB. Enabling them to recognize changes in menstrual bleeding that may increase their risk of iron deficiency diagnosis and seek medical support when required.

When conducting research on iron status in premenopausal females, the following statistical analysis approaches are advised:

1. Employ a generalized linear or non-linear mixed model (see Gałecki and Burzykowski, 2013) or generalized additive mixed model (see Wood, 2017) that suitably estimates the between- and within-participant variability throughout the menstrual cycle by treating participants as a random variable. Given the changes in iron parameters across the menstrual cycle are rarely linear, a spline-based model may provide a more flexible way to analyse their relationship to sex steroid hormone responses.
2. Where multiple OCP brands have been used throughout a study, then if the sample size allows it, the OCP brand may be either fit as a random intercept (in addition to participant ID), and/or a heterogeneous residual variance-covariance structure fit to allow a different, within-participant variance to be estimated per OCP brand.
3. Given the repeated measures nature of menstrual cycle research, autocorrelation should be accounted for by fitting a suitable variance-covariance matrix (e.g., autoregressive, autoregressive moving average, or exponential spatial). In our experience, serum ferritin and hepcidin are often right-skewed, which is often partially addressed *via* log-transformation, or less accurately, by using a non-parametric, rank-based approach (e.g., Wilcoxon signed-rank test). However, ferritin and hepcidin data are often more accurately modeled using a Gamma distribution with a log link function, allowing the response variable to be modeled on its raw unit scale.
4. Baseline serum ferritin level should be included as a covariate in randomized, controlled pre vs. post repeated measures designs to estimate the treatment effect more accurately. We advise against modeling either raw unit or percentage differences in these designs since they do not appropriately account for the dependent nature of the data (see Vickers and Altman, 2001). Serum ferritin is a continuous, zero bound variable that is often unnecessarily categorized into “cut scores” for diagnostic or analysis purposes, whilst this may be attractive from a decision-making perspective, categorization of serum ferritin during statistical modeling may lead to a loss of information, power, and efficiency (Altman, 2014).

de Paula Oliveira et al. (2021) recently employed an excellent statistical approach (i.e., a state-space model) to model the menstrual cycle length in women using a smartphone application to monitor their menstrual symptoms. Future investigations may consider similar statistical approaches to understand how sex steroid hormone fluctuations throughout the menstrual cycle affect iron parameters in female OCP users and non-users.

## Conclusion

Despite active females having a higher risk of developing an iron deficiency than males, only ~11% of iron regulation research has been conducted in female only cohorts, with ~35–40% conducted in male only cohort. Research into iron regulation in females often does not consider the phase of the menstrual cycle (fluctuation in sex hormones), hormonal contraceptive use, or menstrual irregularities. This sex disparity is not exclusive to the research conducted in iron regulation (Costello et al., 2014). In this review, we have provided an in-depth analysis and summary of the current female-specific iron metabolism research, and detailed practical recommendations for iron monitoring, diagnosis, and treatment in female athletes (Figure 1; Table 5). In addition, we have highlighted key areas for future research that are required to better understand iron metabolism in physically active premenopausal females.

**Table 5:** Expected changes to iron parameters throughout the menstrual cycle in active females.

**Commonly measured iron variable**	**What does it measure**	**Early Follicular Phase: Menses**	**Mid to late Follicular phase**	**Luteal phase**	**Factors that will upregulate iron parameters**	**Factors that will downregulate iron parameters**
Serum Iron	Measure of how much iron is in your plasma	Low	Low/ Gradual increase	High	Previous exercise session, exogenous/synthetic estrogens, iron supplements, diurnal variation (afternoon), dietary intake	Menstrual bleeding, diurnal variation (morning), dietary intake, inflammation, E2
Total iron binding capacity (TIBC)	Measure of the blood capacity to bind with iron	High	High	Low	Menstrual bleeding, dietary intake, serum iron, basal iron status	Dietary intake, serum iron, basal iron status
Transferrin Saturation	Measure of a percentage of iron bound to transferrin. Calculated as Serum iron/TIBC	Low	Low/ Gradual increase	High Early luteal phase it is not uncommon for females to present with > 45%	Mirrors changes in serum iron	Mirrors changes in serum iron
Hemoglobin	Measure of free levels of hemoglobin in the blood	Low/ Normal	Increasing/No change	High/ No change	Previous exercise, dehydration, decrease in plasma volume (e.g., post exercise shifts, changes with posture)	Haemodilution, hypovolemia with training or heat adaptation, increase fluid retention due to P4 in luteal phase,
Serum Ferritin	Measure of the body's iron stores	Low/ no change	Increasing/no change	High/no change	Previous exercise, infection/illness, inflammation, iron infusion/injection, prolonged suppression of serum hepcidin	Prolonged elevation in serum hepcidin, iron supplements or iron rich food in iron sufficient individuals
Serum Hepcidin	Measure of the concentration of hepcidin in the blood	Low/No change	Increasing/No change	High/No change	Previous exercise, dehydration, decrease in plasma volume, P4, energy availability status, inflammation/illness/ infection, iron supplements, iron fortified foods, high-normal iron status	Haemodilution, hypovolemia, hydration status, E2, carbohydrate availability, altitude exposure, enhanced erythropoiesis, deficient iron status

## Author contributions

CB and AG conceived the project and drafted the initial manuscript. The work was reviewed and critically reviewed by all authors before all agreed to the final manuscript. All authors contributed to the article and approved the submitted version.

## Conflict of interest

The authors declare that the research was conducted in the absence of any commercial or financial relationships that could be construed as a potential conflict of interest.

## Publisher's note

All claims expressed in this article are solely those of the authors and do not necessarily represent those of their affiliated organizations, or those of the publisher, the editors and the reviewers. Any product that may be evaluated in this article, or claim that may be made by its manufacturer, is not guaranteed or endorsed by the publisher.
